# Effect Sizes of Cognitive and Locomotive Behavior Tests in the 5XFAD-J Mouse Model of Alzheimer’s Disease

**DOI:** 10.3390/ijms242015064

**Published:** 2023-10-11

**Authors:** Moonseok Choi, Hyung-Sup Jang, Taekwon Son, Dongsoo Kim, Young-Jin Youn, Gyu-Bin Hwang, Young Pyo Choi, Yun Ha Jeong

**Affiliations:** 1 Department of Neurodegenerative Diseases Research Group, Korea Brain Research Institute, 61, Cheomdan ro, Dong gu, Daegu 41062, Republic of Korea; moonseok37@kbri.re.kr (M.C.); rapidozzangs@kbri.re.kr (D.K.); yunyj1203@kbri.re.kr (Y.-J.Y.); gyubin2023@kbri.re.kr (G.-B.H.); 2 Laboratory Animal Center, Division of Research Strategy, Korea Brain Research Institute, 61, Cheomdan ro, Dong gu, Daegu 41062, Republic of Korea; jangsimy@kbri.re.kr; 3 Korea Brain Bank, Division of Research Strategy, Korea Brain Research Institute, 61, Cheomdan ro, Dong gu, Daegu 41062, Republic of Korea; taekwon@kbri.re.kr

**Keywords:** 5XFAD-J, Alzheimer’s disease, sample size, behavior, novel object recognition test, LABORAS

## Abstract

Alzheimer’s disease (AD) is characterized by the accumulation of amyloid β (Aβ) plaques in the brain, leading to cognitive impairment and other clinical symptoms. The 5XFAD mouse model is commonly used in AD research because it expresses five human transgenes that result in the accumulation of Aβ plaques and cognitive decline at a relatively early age. Behavioral experiments are frequently conducted using this model; however, the effect size has not yet been reported. In this study, we examined basic cognition and locomotion in 5XFAD mice with a C57BL6/J background (5XFAD-J) at 6 months of age, a period in which impairments of cognitive function and locomotion are commonly observed. We analyzed the effect sizes of cognitive and locomotive experiments in the 5XFAD mice compared with those in the wild-type mice. Our results suggest that for long-term memory analysis, the novel object recognition test (*p* = 0.013, effect size 1.24) required a sample size of at least 12 to obtain meaningful results. Moreover, analysis of general locomotion over total distance with the Laboratory Animal Behavior Observation, Registration and Analysis System (LABORAS) test during the dark phase (*p* = 0.007, effect size −1.37) needed a sample size of 10 for a statistical power (1-β) of 0.8. In conclusion, we can conduct more ethical and scientifically rigorous animal experiments using 5XFAD mice based on the effect and sample sizes suggested in this study.

## 1. Introduction

Alzheimer’s disease (AD) is a neurodegenerative disorder characterized by progressive loss of cognitive function, including memory, language, and executive functions [1]. It is the most common cause of dementia. It affects millions of people globally [2]. This disease is characterized by the presence of amyloid plaques caused by the accumulation of the amyloid-β (Aβ) peptide, neurofibrillary tangles through the hyperphosphorylation of tau protein, neuronal loss, synaptic dysfunction, and altered long-term potentiation and depression in the brain [3].

AD animal models, such as 5XFAD, APP/PS1, and P301S, have been developed to study the disease’s pathological mechanisms and test potential treatments [4]. Among these models, the 5XFAD model expresses five human transgenes with the Thy1 promoter expressed through neuronal cells, including the amyloid precursor protein (APP) carrying the K670N_M671L (Swedish), I716V (Florida), and V717I (London) mutations, as well as presenilin (PSEN1) carrying the M146L and L286V mutations. These mutations result in the accumulation of Aβ plaques, which are the pathological hallmarks of AD [5]. The 5XFAD transgenic (Tg) mouse model was initially developed using the B6SJLF1/J mouse strain (5XFAD-SJL). However, the maintenance of breeding pairs in this strain is complicated. A new strain of 5XFAD mice was developed from the C57B6-J strain (5XFAD-J) to overcome this issue, enabling more efficient breeding and thus facilitating AD-related research using this model [6]. The 5XFAD-J model has been used extensively to understand AD pathogenesis, including age-dependent cognitive deficits. Notably, several behavioral experiments have been developed to assess cognitive impairment and general locomotive behavior in AD animal models. These include the Y-maze test for short-term spatial memory, the novel object recognition (NOR) test for long-term recognition memory, and the Laboratory Animal Behavior Observation, Registration and Analysis System (LABORAS) test for measuring general locomotion, such as motor activity and repetitive behavior [7,8]. These experiments provide valuable insights into AD progression and potential treatments and are widely used in AD animal models, such as 5XFAD-J and 5XFAD-SJL mice. However, it should be noted that differences in genetic background and environmental factors can result in different outcomes for each behavioral experiment. Therefore, specific animal models, experimental conditions, and sample sizes should be carefully considered when designing and interpreting behavioral experiments.

In statistics, effect size is a quantitative measure of the magnitude of the difference or relationship observed between groups or variables in a study. It represents the size of the difference in outcomes, such as physiological measurements and behavioral responses, between experimental and control groups, and can be used to determine the sample size for studies or to examine effects across studies. Sample size refers to the number of animals for each group included in a study, and is crucial for the validity and reliability of results in animal models [9]. Hence, this study focused on the optimal sample size and behavioral characteristics of the 5XFAD-J model, as well as its strengths and limitations in studying cognitive impairment and other behaviors.

## 2. Results

In brief, the experimental protocol involved a sequential assessment of general locomotion using the LABORAS test, followed by an evaluation of cognitive impairment using the Y-maze and NOR tests at 6 and 9 months. Our study utilized 10 male WT and 5XFAD-J (hereafter referred to as 5XFAD) mice per group for behavioral experiments using the same mice from each group at 6 and 9 months.

**Figure 1:**
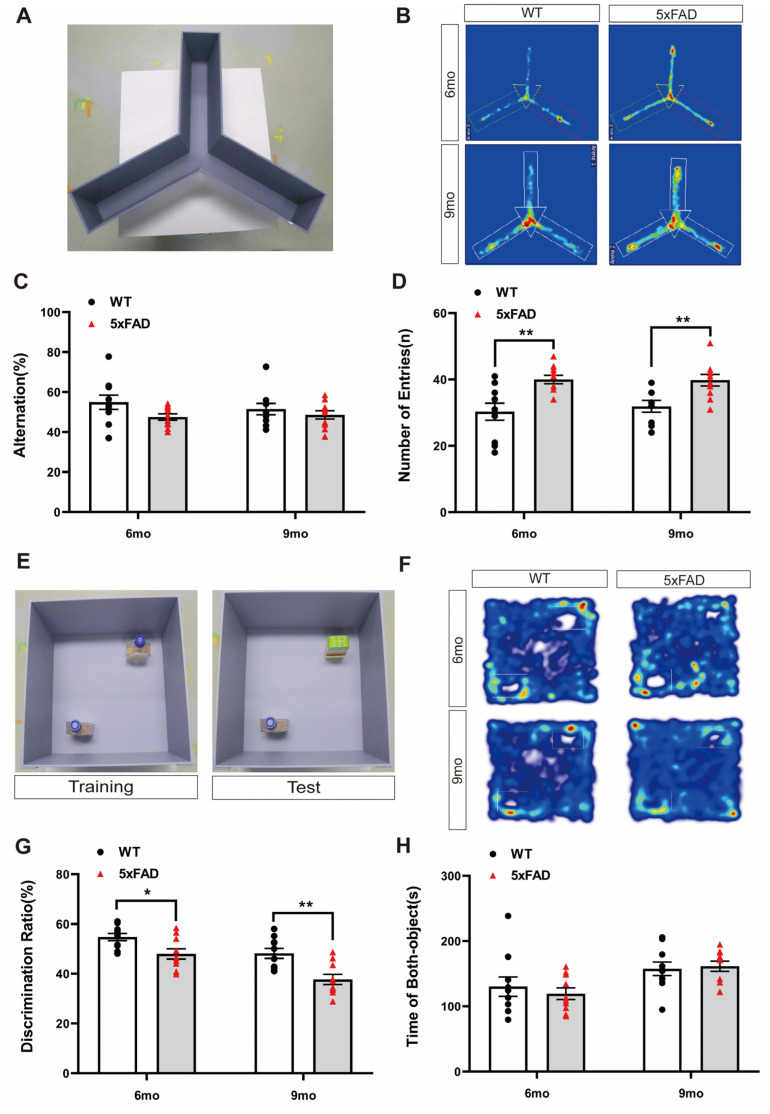
Exploration of cognitive behavioral experiments in 6- and 9-month-old 5XFAD mice. (**A**) Representative photograph of the Y-maze test. (**B**) Representative trajectory heatmap images of mouse positions in the experimental arena. (**C**) The percentage of alternation in the Y-maze test did not differ significantly between groups. (**D**) The number of entries in the Y-maze test was higher than that for the WT mice in the 6- and 9-month-old 5XFAD mice. (**E**) Representative photograph of the NOR test. (**F**) Representative trajectory heatmap images of mouse positions in the experimental arena. (**G**) The percentage of discrimination ratio in the NOR test was lower than that for the WT mice. (**H**) The time spent with both objects in the NOR test did not differ. *n* = 10 per group. * *p* < 0.05, and ** *p* < 0.01 (*t*-test). Data are presented as the mean ± standard error of the mean. WT, wild-type; NOR, novel object recognition; mo, month.

To assess short-term memory impairment in a mouse model of AD, we performed the Y-maze test with WT and 5XFAD mice. The Y-maze test was conducted according to the depicted protocol, and the trajectory heatmap images of each group during the behavior analysis revealed a potential for hyperactivation in the 5XFAD group (Figure 1A,B). The alternation ratio of each arm and the number of arm entries were recorded. The percentage of spontaneous alternations was lower, but not significantly, in the 5XFAD mice than in the WT mice at both ages (Figure 1C). However, the number of entries was significantly higher in the 5XFAD mice than in the WT mice at both ages (Figure 1D). These results suggest that Y-maze behavioral analysis is inappropriate for validating the decline in short-term memory in 5XFAD mice regardless of age because 5XFAD mice exhibit Y-maze performance without a notable decline in memory, along with hyperactivity. This hyperactivity, an indicator of anxiety-like behavior, may have influenced the behavior outcomes in the Y-maze.

To assess long-term memory impairment in a mouse model of AD, we performed the NOR test. The NOR test was conducted according to the depicted protocol, and the trajectory heatmap images of each group during the behavior analysis revealed a potential for decreasing novel object interaction in the 5XFAD group (Figure 1E,F). The discrimination ratio was significantly lower in the 5XFAD mice than in the WT mice at both ages (Figure 1G). However, the total exploration time of both objects did not differ between the 5XFAD and WT mice at either age (Figure 1H). These results indicate that the 5XFAD group exhibited a significantly reduced discrimination ratio for distinguishing novel objects compared to the WT group in the NOR test. This suggests that the NOR test is suitable when investigating changes in long-term memory in 5XFAD mice. Unlike the previous Y-maze results, it is possible to confirm the impairment of long-term memory through the NOR test even in the presence of hyperactivity.

**Figure 2:**
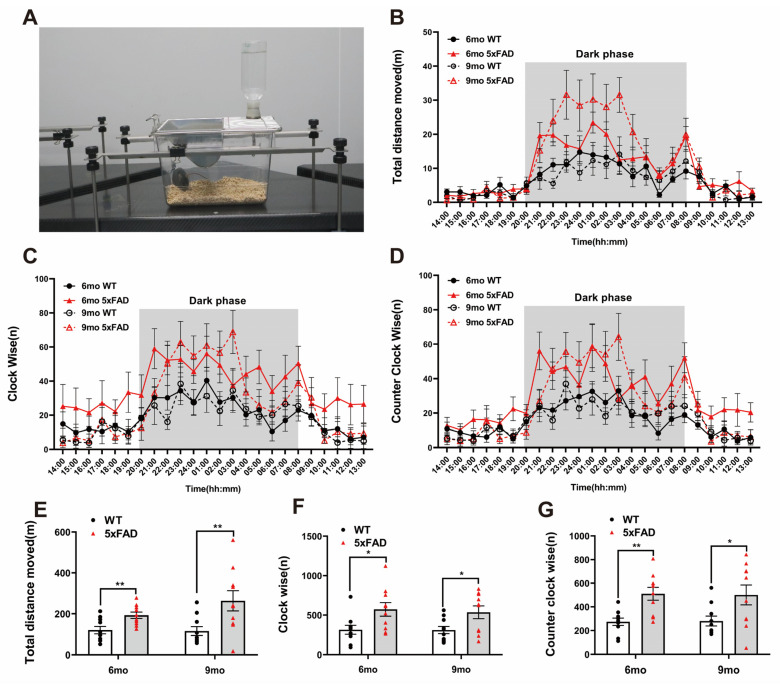
Exploration of hyperactivity and circling behavioral experiments in 6- and 9-month-old 5XFAD mice. (**A**) Representative photograph of the Laboratory Animal Behavior Observation, Registration and Analysis System (LABORAS) test. (**B**–**D**) Representative line graph of the analyzed total distance and circling by hours during the test day. (**E**) The total distance traveled by the 5XFAD mice increased more during the dark phase than the WT mice in the LABORAS test. (**F**) Clockwise circling was higher in both the 6- and 9-month-old 5XFAD mice during the dark phase than the WT mice in the LABORAS test. (**G**) The amount of counterclockwise circling was higher in both the 6- and 9-month-old 5XFAD mice during the dark phase than the WT mice in the LABORAS test. *n* = 10 per group. * *p* < 0.05, and ** *p* < 0.01 (*t*-test). Data are presented as the mean ± standard error of the mean. WT, wild-type; LABORAS, Laboratory Animal Behavior Observation, Registration and Analysis System; mo, month.

To assess motor activity and repetitive behavior at night (dark phase), we performed the LABORAS test. The LABORAS test was conducted according to the depicted protocol (Figure 2A). Both the WT and 5XFAD groups displayed significantly increased activity and circling in the dark phase (Figure 2B–D). The traveled distance was significantly higher in the 5XFAD mice than in the WT mice at both ages (Figure 2E). Clockwise (CW) and counterclockwise (CCW) circling occurred significantly more frequently in the 5XFAD mice than in the WT mice at both ages (Figure 2F,G). These results indicate that the 5XFAD mice exhibited heightened locomotive behavior such as traveled distance and circling, which may reflect hyperactivity or repetitive behaviors associated with anxiety-like behavior. LABORAS behavioral analysis is considered appropriate for validating the increased hyperactivity and circling in 5XFAD mice. The assessment of long-term memory through the NOR test and the measurement of hyperactivity and repetitive behavior using the LABORAS test can be proposed as significant indicators in AD drug screening tests. These results suggest that 5XFAD mice can be considered a valuable model for the behavioral validation of potential AD therapeutics from the perspective of cognition and activity.

In addition to statistical considerations, ethical principles and animal welfare regulations should be considered when determining the sample size for experiments involving animal models of AD. Using animals in research must be justified and minimized to reduce the number of animals required to achieve the research goals [10]. The selection of an appropriate sample size should balance the study’s scientific value with ethical considerations and the potential impact on animal welfare. Moreover, when selecting the number of mice required for an investigation, the possible loss of data due to experimental errors or animal mortality should be considered to ensure that the research is efficient and cost-effective. Overall, careful consideration of statistical and ethical factors is crucial for determining the appropriate sample size for experiments involving the 5XFAD mouse model of AD. Our findings suggest that the effect size, sample size, and statistical power of behavioral experiments in the 5XFAD model can vary depending on the specific behaviors measured and the age of the mice. Careful consideration of these factors is essential when designing experiments to ensure reliable results. Table 1 summarizes the effect and sample sizes required for each behavioral analysis. Furthermore, we assessed the changes in amyloid plaques and protein expression such as Aβ, β-secretase 1 (BACE1), presenilin enhancer 2 (PEN2), synaptophysin, and glial fibrillary acidic protein (GFAP) in the hippocampus of 6-month-old 5XFAD mice (Figure S1), and we also obtained the effect and sample sizes in each pathological analysis, like the behavioral analysis (Table S1).

Based on the sample size analysis results, a sample size of >20 individuals per group should be used for the percentage of alternation triads in the Y-maze test at 6 months of age (*p* = 0.074, effect size 0.849) and at a statistical power (1-β) of 0.8. However, significant results may be difficult to obtain at 9 months of age (*p* = 0.422, effect size = 0.368), with a sample size of 117 for a power of 0.8. Conversely, the number of entries in the Y-maze test at 6 months (*p* = 0.005, effect size −1.51) required a sample size of 8 for a power of 0.8, indicating possible hyperactivity in the 5XFAD mice (Table 1). These results suggest that careful consideration of the sample size and potential confounding factors, such as hyperactivity, is necessary when conducting behavioral experiments using the 5XFAD mouse model.

The percentage of discrimination in the NOR test showed a significant difference at 6 months of age (*p* = 0.013, effect size 1.24), requiring at least 12 individuals per group to obtain meaningful results. The significant difference further increased at 9 months of age (*p* = 0.002, effect size 1.61), with a sample size of 8 for a power of 0.8. However, the total exploration time for each object was not significantly different at 6 or 9 months of age (Table 1). These results suggest that the NOR test is a highly efficient behavioral analysis method for assessing long-term memory in 5XFAD mice regardless of age from 6 to 9 months.

Analysis of general locomotion over total distance with the LABORAS test during the dark phase showed significant differences at 6 months of age (*p* = 0.007, effect size −1.37), requiring 10 individuals per group for a power of 0.8. Similar results were obtained at 9 months (*p* = 0.018, effect size −1.23), requiring 12 individuals for a power of 0.8. Repetitive behavioral analyses using CW circling during the dark phase showed significant differences at 6 months of age (*p* = 0.022, effect size −1.12), requiring 14 individuals per group for a power of 0.8; similar results were obtained at 9 months (*p* = 0.029, effect size −1.08), requiring 15 individuals for a power of 0.8. Furthermore, CCW circling during the dark phase showed significant differences at 6 months of age (*p* = 0.001, effect size −1.69), requiring 7 individuals per group for a power of 0.8; similar results were obtained at 9 months (*p* = 0.036, effect size −1.05), requiring 16 individuals for a power of 0.8 (Table 1). Therefore, hyperactivity and repetitive behavior analysis using the LABORAS test can be used as an efficient behavioral analysis method at 6 and 9 months.

## 3. Discussion

Various behavioral experiments, including the Y-maze, NOR test, and LABORAS test, have been utilized to assess cognitive impairment, general locomotion, and repetitive behavior in animal models of AD [11]. In addition to the Y-maze, NOR test, and LABORAS test, other tests such as the T-maze, Morris water maze (MWM), contextual fear conditioning test, rotarod, elevated plus maze, eight-arm radial maze, and Barnes maze are also commonly used in AD research [11,12,13,14]. The effect size, sample size, and statistical power of behavioral experiments in the 5XFAD model can differ depending on the specific behavior being measured and the age of the mice. Therefore, carefully considering these factors is crucial when designing experiments to ensure reliable results. A power analysis was conducted to determine the appropriate sample size for each experiment. By carefully selecting an appropriate sample size using power analysis, researchers can ensure the scientific value of their studies while respecting ethical considerations and animal welfare regulations [15]. The power analysis method chooses the necessary sample size to reach the specified power to detect a scientifically meaningful difference between groups. In statistics and experimental design, Cohen’s d is a measure of effect size that quantifies the difference between the means of two groups in terms of standard deviation units. It is commonly calculated to assess the practical significance of an observed difference between groups, helping to determine if a finding is not only statistically significant but also practically meaningful. In animal studies, a Cohen’s d of 0.8 or 0.9 indicates a large effect size, suggesting a substantial and noteworthy difference between the groups being compared [9]. Our results suggest that the sample sizes required to investigate the impairment of short-term memory in Y-maze analysis involving 5XFAD mice needs to exceed 20 per group to acquire meaningful differences. In contrast, the results in this study indicate that a sample size of around 10 mice per group is enough to detect significant differences in long-term recognition memory and general locomotion by the NOR and LABORAS tests in 5XFAD mice.

Notably, previous studies have analyzed various behavioral characteristics, such as cognitive impairment, anxiety, and motor function in AD mouse models [11]. Deterioration of cognitive ability, effect size, and sample size have been analyzed through MWM behavioral analysis according to age and sex using TG-2576 mice. The effect only becomes apparent between 12 and 18 months and requires a large sample size [16]. Moreover, the sample size varies according to the sound–cue interval in the behavioral analysis of Contextual and Trace Fear Conditioning in the P301S tau mouse model [17]. A recent study suggesting sample and effect sizes for behavioral and pathological characteristics with 4- to 6-month-old 5XFAD-SJL AD model mice showed no alteration in cognitive impairment by the NOR and Y-maze tests [18]. In addition, the MWM behavior test showed no alteration in spatial learning between training and probe days, while cognitive function was decreased during the reverse learning task in the 5XFAD group [18]. However, few studies in the neuroscience field have analyzed the sample size for each behavioral analysis using the effect size in animal models.

The current study highlights the importance of presenting the effect size and sample size per group for the cognitive and locomotive behaviors of 5XFAD mice, which are widely used in the non-clinical research of AD-related pathology. This approach makes animal experiments more ethical and feasible in related fields by suggesting an appropriate effect size and sample size.

## 4. Materials and Methods

### 4.1. Animals

B6.Cg-Tg(APPSwFlLon,PSEN1*M146L*L286V)6799Vas/Mmjax (5XFAD-J) mice were purchased from The Jackson Laboratory (Bar Harbor, ME, USA; MMRRC Stock #34848). These 5XFAD mice, expressing human APP670, 671sew, 716Flo, 717Lon, and PSEN1M146L, L286V mutations, are the most widely used AD mouse models. Non-transgenic WT littermates used as control mice in this study were also purchased from The Jackson Laboratory. The animals were housed in a controlled environment with a 12 h light/dark cycle and maintained at a consistent temperature. The behavioral tests were performed in the Laboratory Animal center at the Korea Brain Research Institute.

### 4.2. Behavioral Experiments

#### 4.2.1. Y-Maze Test

We performed the Y-maze test to test short-term spatial memory as previously described [19]. The Y-maze was composed of three equally sized arms made of a gray acrylic plate with dimensions of 4.5 cm × 30 cm × 13 cm. The mice were placed in the test room for 1 h for habituation. The Y-maze was then cleaned with 70% ethanol. Following habituation, the mice were placed in the center of the Y-maze and allowed to explore freely for 5 min. SMART video tracking software version 3.0 was used to record the video footage. The alternation ratio of each arm and the number of arm entries were recorded.

#### 4.2.2. Novel Object Recognition Test

We performed the NOR test, a widely employed mouse behavioral analysis designed to assess alterations in long-term memory [7]. Briefly, the task mice were positioned in a gray acrylic chamber measuring 40 cm × 40 cm × 25 cm to assess long-term memory. The mice were allowed to habituate to the test environment for 1 h, following which the chamber was sanitized with 70% ethanol. During habituation, two identical objects (cell flasks) were placed diagonally at opposite corners of the test chamber, and the mice were allowed to explore them for 10 min. After 24 h, on the test day, the mice were placed back into the chamber and allowed to explore a novel object (a LEGO block) and a familiar object (a cell flask) placed at diagonally opposite corners for 10 min. EthoVision XT 14 video tracking software (Noldus, Wageningen, The Netherlands) was used to record the experiments. The exploration time for each object was analyzed.

#### 4.2.3. LABORAS Test

We performed the LABORAS test as previously described [19]. After weighing, the mice were placed in the LABORAS bedded home cage. The LABORAS test was carried out for 72 h from 1:00 p.m. Both groups were given a normal chow diet and water for the test duration. The system automatically monitored and recorded a range of basic mouse behaviors, including distance traveled, rearing, climbing, and circling, by tracking the movement of the mice. Data measured during the last 24 h were shown on an hourly basis. For the dark-phase analysis, data obtained during the last 12 h dark phase (20:00 to 08:00) were used for the comparative analysis between groups.

### 4.3. Sample Size Analysis

Effect and sample sizes were calculated using Jamovi software (version 2.3.1) [20]. Cohen’s d statistic was calculated to compare the effect sizes [21] for the behavioral experiments between the two groups at 6 and 9 months. For the two-group t-test, we specified the means (μ1, μ2) and the common standard deviation (σ) in the groups to calculate Cohen’s d = |μ1 − μ2|/σ [9]. To conduct a power analysis, the effect size was used to calculate the sample size required for statistical power, with 1-β of 0.8 and a type 1 error (α) set at 0.05.

### 4.4. Statistical Analysis

The statistical analysis was conducted using GraphPad Prism 9 (GraphPad Software, San Diego, CA, USA) and Jamovi. The presented data were analyzed using Student’s *t*-test, Welch’s t-test, or the Mann–Whitney U test, depending on normality and equal variance. Statistical significance was set at *p* < 0.05. All data are presented as means ± standard errors.

## Figures and Tables

**Table 1:** Effect and sample size of the WT mice vs. the 5XFAD mice in the behavior test.

Genotype WT/5XFAD (n)	Behavior	Analysis	6-Month-Old					9-Month-Old				
			WT Mean (SD)	*p*-Value	Effect Size (Cohen’s d)	Sample Size per Group		WT Mean (SD)	*p*-Value	Effect Size (Cohen’s d)	Sample Size per Group	
			5XFAD Mean (SD)			(1-β) = 0.8	(1-β) = 0.9	5XFAD Mean (SD)			(1-β) = 0.8	(1-β) = 0.9
10/10	Y-maze	Alternation triad (%)	54.9 (11.2)	0.074	0.849	23	31	51.5 (9.0)	0.422	0.368	117	157
			47.5 (5.1)					48.6 (6.6)				
		Number of entries (n)	30.3 (8.1)	0.005 ^#^	−1.51	8	11	31.9 (5.6)	0.005	−1.41	9	12
			40.0 (4.0)					39.8 (5.6)				
10/10	NOR	Discrimination (%)	54.8 (4.6)	0.013	1.24	12	15	48.2 (6.3)	0.002	1.61	8	10
			48.0 (6.5)					37.7 (6.5)				
		Time of both objects (sec)	130.2 (46.7)	0.912 *	N.A	N.A		157.7 (33.0)	0.76	−0.138	826	1105
			119.5 (27.8)					161.7 (25.0)				
10/10	LABORAS (Dark phase)	Total distance (m)	120.3 (57.0)	0.007	−1.37	10	13	115.6 (68.6)	0.018 ^#^	−1.23	12	15
			192.8 (49.0)					263.6 (156.3)				
		Clockwise circling (n)	314.3 (179.3)	0.022	−1.12	14	18	310.6 (146.7)	0.029 ^#^	−1.08	15	20
			572.1 (271.4)					535.2 (254.1)				
		Counterclockwise circling (n)	273.6 (97.7)	0.001	−1.69	7	9	279.9 (131.1)	0.036 ^#^	−1.05	16	21
			510.7 (173.0)					500.3 (267.6)				

*: Mann–Whitney (non-normal distribution of the data), ^#^: Welch’s (unequal variances between the groups), NOR; novel object recognition, LABORAS; Laboratory Animal Behavior Observation, Registration and Analysis System, N.A: not available.

## Data Availability

All original data for this study are presented within the paper and are available from the corresponding author upon reasonable request.

## Supplementary Materials

The following supporting information can be downloaded at: https://www.mdpi.com/article/10.3390/ijms242015064/s1.
