# Supplementary material for: Effect Sizes of Cognitive and Locomotive Behavior Tests in the 5XFAD-J Mouse Model of Alzheimer’s Disease
Source: Int J Mol Sci. 2023 Oct 11;24(20):15064. doi: 10.3390/ijms242015064 (PMC10606448; doi:10.3390/ijms242015064)
Supplement: Supplementary file 1 [file ijms-24-15064-s001.zip › ijms-2600763-supplementary.pdf]

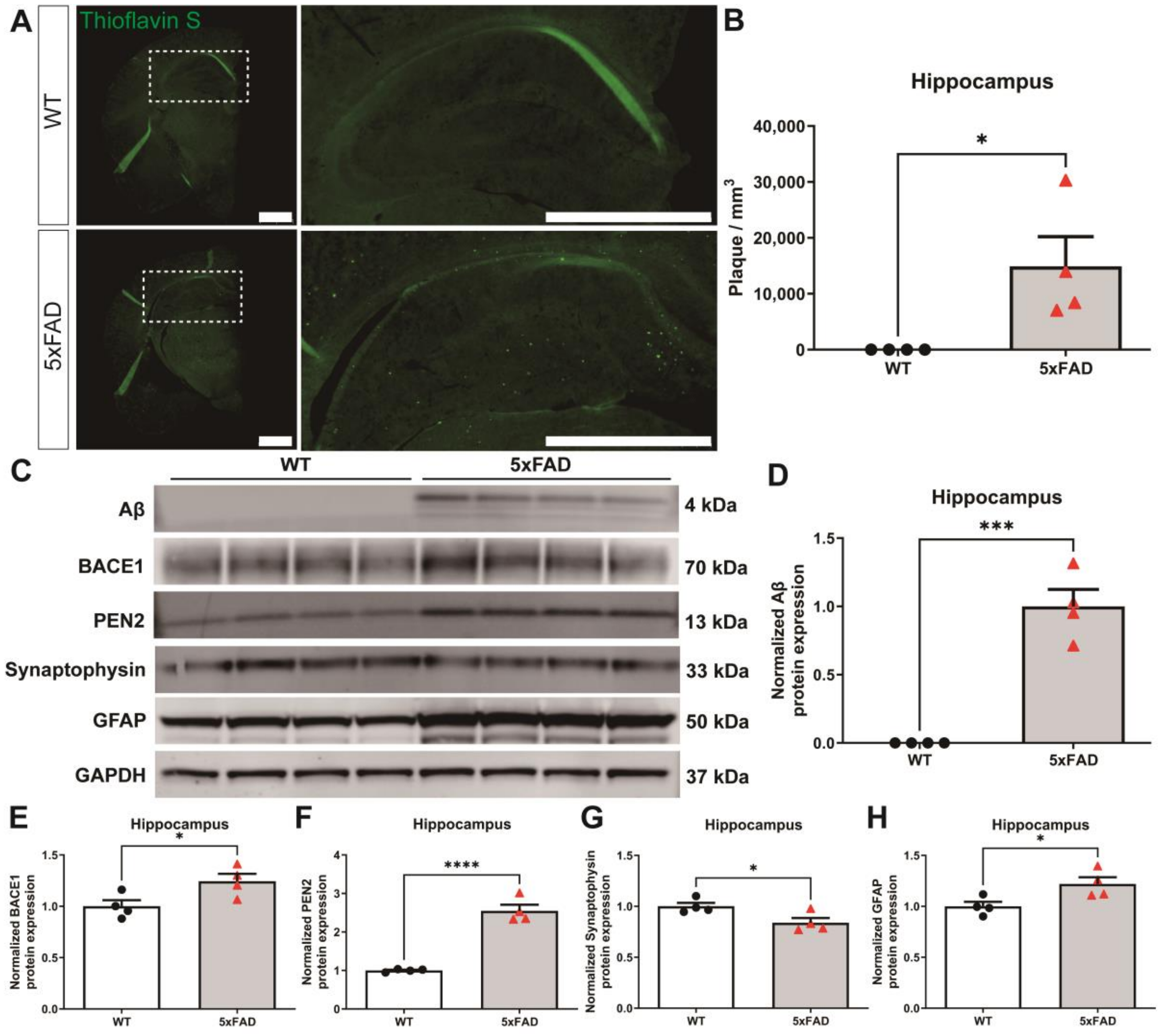

Figure S1. Pathological analysis in 6-month-old 5XFAD mice. (A) Representative images of thioflavin-S staining of the hippocampus. Scale bars = 1 mm. (B) Bar graph of amyloid plaque numbers in the hippocampus. n = 4 per group. (C) Representative western blot images of hippocampal proteins. (D-H) Bar graph of protein expression in the hippocampus. n = 4 per group. * p<0.05, *** p<0.001, **** p<0.0001, (t-test). Data are presented as mean ± standard error of the mean. WT, wildtype; Aβ, amyloid β peptide; BACE1, β-secretase 1; PEN2, presenilin enhancer 2; GFAP, glial fibrillary acidic protein; GAPDH, Glyceraldehyde 3-phosphate dehydrogenase; kDa, kilodalton

Table S1. Effect size and sample size of WT vs. 5XFAD mice in the pathological analysis.

**Genotype WT/5XFAD (n)**	**Molecular**	**Analysis**	**6-month-old**				
			**WT Mean (SD)**<br>**5XFAD Mean (SD)**	***P* value**	**Effect size (Cohen's d)**	**Sample size per group** (1-β) = 0.8	(1-β) = 0.9
4/4	Thioflavin-S	Number of plaque (/mm^3)	0 (0.00)<br>14,868 (10,675)	0.032	1.97	6	7
4/4	Aβ	Protein expression	0 (0.00)<br>1 (0.25)	<0.001	-5.70	≤3*	3
4/4	BACE1	Protein expression	1.00 (0.12)<br>1.24 (0.15)	0.042	-1.82	6	8
4/4	PEN2	Protein expression	1.00 (0.04)<br>2.55 (0.32)	<0.0001	-6.81	≤3*	≤3*
4/4	Synaptophysin	Protein expression	1.00 (0.07)<br>0.84 (0.09)	0.033	1.94	6	7
4/4	GFAP	Protein expression	1.00 (0.09)<br>1.22 (0.14)	0.035	-1.91	6	7

*: Sample size is less than 3; The number of subjects was three to ensure minimal statistical significance. Aβ, Amyloid β peptide; BACE1, β-secretase 1; PEN2, presenilin enhancer 2; GFAP, Glial fibrillary acidic protein

Supplementary Materials and Methods

1. Sampling

Anesthesia was induced in mice via intraperitoneal injection of 2,2,2-tribromoethanol (150 mg/kg, Avertin, Sigma, St. Louis, MO, USA). Serial perfusion was performed using 1× phosphate-buffered saline (PBS, pH 7.2) for 5 minutes. Brain tissue samples were then collected from the mice followed by fixation of the brain with 4% paraformaldehyde for Thioflavin S staining.

2. Thioflavin S staining

To assess amyloid plaques, brain tissues were subjected to Thioflavin S staining. Briefly, brain sections were washed 3 times with 1× PBS and subsequently incubated with a 1% Thioflavin S solution (50% ethanol in 1× PBS) for 8 minutes at 24°C. The stained samples were then rinsed twice with 85% ethanol for 5 minutes, followed by a 5-minute wash in 95% ethanol and a triple wash in 1× PBS. The stained brain tissue samples were imaged using Panoramic Slide Scan, and amyloid plaque quantification was conducted using Stereo Investigator (MBF Bioscience, VT, USA).

3. Western blot

For protein expression analysis, hippocampal tissues were homogenized utilizing RIPA solution supplemented with a protease and phosphatase inhibitor cocktail. Protein samples extracted from the hippocampus tissues were quantified using the BCA method. Equal quantities (20 μg) of the protein samples were loaded, mixed with 4× protein sample buffer, and subjected to electrophoresis on pre-cast 4%–12% polyacrylamide gels using an Invitrogen western blot system (Invitrogen, MA, USA) at 150 V for 40 minutes. Subsequently, the separated proteins were transferred onto polyvinylidene fluoride (catalog no. LC2002, Novex, Hochdorf, Switzerland) or nitrocellulose (catalog no. LC2000, Novex) membranes at 20 V for 2 hours. Primary antibodies, including those against BACE1 (rabbit, 1:1,000, # 5606, Cell Signaling Technology), PEN2 (rabbit, 1:1,000, # 8598, Cell Signaling Technology), Aβ (6E10) (mouse, 1:2,000, # 803001, BioLegend, CA, USA), synaptophysin (rabbit, 1:1,000, # SAB 450-2906, Sigma, MO, USA), and GFAP (rabbit, 1:2,000, # Z0334, Dako, Glostrup, Denmark), were incubated with the membranes overnight at 4°C. Following membrane washing using Tris-buffered saline with 0.3% Tween 20 Detergent (TBS-T), secondary antibodies including IRD-800 anti-rabbit (donkey, 1:2,000, # 926-32213, LI-COR, NE, USA) and IRD-800 anti-mouse (donkey, 1:5,000, # 926-32212, LI-COR) were applied to the membranes for 2 hours at 24°C. After subsequent membrane washing 3 times with 1× PBS, fluorescence signals were detected and analyzed using Odyssey-CLx (LI-COR, NE, USA).
